# Research self-efficacy in early-career pharmacists: Tool validation and correlation with personal attributes

**DOI:** 10.1016/j.rcsop.2025.100693

**Published:** 2025-12-06

**Authors:** Aline Hajj, Hala Sacre, Chadia Haddad, Jenny Elia, Joya El Ghawi, Lina Haidar, Lama Dimachkieh, Mahmoud Nasrallah, Jihan Safwan, Deema Rahme, Sukaina Basma, Salameh Pascale

**Affiliations:** a Institut National de Santé Publique, d'Épidémiologie Clinique et de Toxicologie-Liban (INSPECT-LB), Beirut, Lebanon; b Faculté de Pharmacie, Université Laval, Québec, Canada; c Oncology Division, CHU de Québec Université Laval Research Center, Québec, Canada; d Research Department, Psychiatric Hospital of the Cross, Jal Eddib, Lebanon; e Faculty of Public Health, Lebanese University, Fanar, Lebanon; f Lebanese Pharmacy Students' Association, Beirut, Lebanon; g School of Pharmacy, Lebanese International University, Beirut, Lebanon; h Pharmacy Practice Department, Faculty of Pharmacy, Beirut Arab University, Beirut, Lebanon; i Faculty of Pharmacy, Lebanese University, Hadat, Lebanon; j Gilbert and Rose-Marie Chagoury School of Medicine, Lebanese American University, Beirut, Lebanon; k Department of Primary Care and Population Health, University of Nicosia Medical School, 2417 Nicosia, Cyprus

**Keywords:** Pharmacy, Research, Research self-efficacy, Global self-efficacy, Strategic thinking, Leadership, Education

## Abstract

**Introduction:**

Self-efficacy in research and personal characteristics of pharmacists are necessary to lead and implement pharmaceutical research strategies. This study primarily aimed to confirm the validity of the Research Self-Efficacy Scale (RSES) among early-career pharmacists; a secondary objective was to assess participants' perspectives on research self-efficacy while considering personal characteristics, such as strategic thinking and leadership.

**Methods:**

Using an exploratory factor analysis and internal consistency measure, the RSES scale validity and reliability were assessed among Lebanese early-career pharmacists. Its association with personal attributes, such as global self-efficacy, leadership, and strategic thinking, was also assessed through correlation with validated measures.

**Results:**

The RSES scale was found to be valid and reliable. Pharmacists from foreign universities scored higher on the RSES compared to their counterparts from Lebanese institutions. Additionally, a positive correlation was found between self-efficacy, generalized self-efficacy, and strategic thinking scores, while differences between universities and year of study did not reach statistical significance.

**Conclusion:**

This study demonstrated that the RSES is a valid and reliable tool for assessing research self-efficacy among early-career pharmacists in Lebanon. Several strategies could be implemented at the institutional and national levels to strengthen self-efficacy and cultivate a sustainable research environment. These include enhancing educational frameworks, integrating research opportunities, and fostering international collaborations. Future research using this validated scale will be instrumental in evaluating the effectiveness of such interventions.

## 1 Introduction

Pharmacy, like any health profession, needs its three pillars to advance: research, education, and practice, with pharmacists' involvement in research being essential. Hence, students and early-career pharmacists should develop research self-efficacy early on,^1^^,^^2^ as research self-efficacy is a critical determinant of pharmacy students' engagement with scholarly activities and their subsequent research involvement and professional development. This concept refers to an individual's confidence in their ability to overcome challenges and sustain efforts in addressing significant problems.^3^ Evidence indicates that self-efficacy is positively influenced by academic support, experiential learning, and structured research education, which collectively reduce research-related anxiety and foster greater confidence in research tasks.^4^ These factors are essential for supporting researchers, who often complain about curricular overload, competing academic demands, and institutional constraints such as limited protected time and resources for research participation.^5^

Moreover, becoming an efficient researcher is not solely defined by technical expertise but also by developing a complex array of critical skills and competencies. Consequently, research self-efficacy in pharmacy is closely linked to personal characteristics such as leadership, general self-efficacy, and strategic thinking.^6^^,^^7^ Pharmacists with higher levels of general self-efficacy tend to be more confident in conducting research and engaging in patient-centered roles, enhancing their contributions to the healthcare system.^2^^,^^8^ Leadership skills, in particular, are essential to improving research self-efficacy, as pharmacists with strong leadership qualities are better equipped to manage research complexities, collaborate with interdisciplinary teams, and address challenges effectively.^2^^,^^9^ Furthermore, leadership within the pharmacy profession fosters the initiative to pursue research opportunities and mentor others, reinforcing a culture of continuous learning, innovation,^10^ and health system improvement.^11^ Similarly, strategic thinking significantly influences research self-efficacy.^3^^,^^12^ Pharmacists with strategic thinking skills typically approach research projects with meticulous planning—anticipating potential challenges and developing innovative solutions—which enhances their confidence and effectiveness in research. This competency enables the achievement of research goals while aligning research activities with the evolving needs of pharmacy practice, maximizing the relevance and impact of work.^13^^,^^14^

In Lebanon, a low-income country in the Middle East, the pharmaceutical sector faces persistent challenges in pharmacy practice and education, exacerbated by the lack of a cohesive national health research policy. This policy gap has led to fragmented, redundant, and often discontinuous research efforts,^15^ with pharmacists rarely involved in research due to multiple barriers,^16^ including limited exposure to research activities, lack of structured research training, insufficient mentorship, and high levels of research-related anxiety. To address these issues, the scientific committee at the Order of Pharmacists of Lebanon (OPL) has developed a National Pharmaceutical Strategy in collaboration with academic researchers and other stakeholders in Lebanon.^17^ It also suggested strategic recommendations for education, workforce, and research.^15^^,^^18^ However, these strategies could not be implemented due to the ongoing economic crisis and the impact of regional conflicts.^19^ Consequently, researchers with leadership attributes, a commitment to serving the community and profession, and the ability to apply strategic thinking in policymaking are essential to drive these endeavors forward.

The establishment and implementation of a comprehensive National Pharmaceutical Research Strategy are essential to align research priorities with healthcare needs, promote collaboration among institutions, and optimize resource utilization in Lebanon.^15^^,^^17^ The critical steps toward achieving these objectives include strengthening the research culture within educational institutions, enhancing interinstitutional and interprofessional collaborations, and building research capacity among pharmaceutical professionals.^15^ Addressing these challenges through innovative solutions and strategic planning could position Lebanon as a regional hub for pharmaceutical research, potentially contributing to improved patient care and public health outcomes.^15^^,^^17^

While education is pivotal to preparing future researchers and research leaders, little is known about early-career pharmacists' preparation for research. Lebanon's universities and hospitals are involved in pharmaceutical research education, with five institutions offering pharmacy programs and postgraduate research opportunities in several pharmaceutical fields.^18^ Integrating research competencies into pharmacy curricula, guided by a validated academic framework, is essential to equip the next generation of researchers with the necessary skills and knowledge.^15^^,^^18^ Educating researchers on leadership and policymaking is also crucial. Studies have demonstrated the importance of a multidisciplinary approach to research education, fostering active student participation in research activities and acting later in shaping research strategies. This approach would also bridge the gap between academic training and optimized practice.^15^^,^^18^^,^^19^ Therefore, the relationship between pharmacists' characteristics and their research success in academic settings has still to be explored. Addressing this gap is crucial for understanding how leadership, self-efficacy, and strategic thinking influence pharmacists' research capabilities and career trajectories; this work is all the more appealing in the absence of locally validated scales for research self-efficacy assessment.

This study primarily aimed to assess the validity and reliability of the Research Self-Efficacy Scale (RSES) among early-career pharmacists (pharmacy students and recent graduates); a secondary objective was to examine participants' perspectives on research self-efficacy while considering personal characteristics, such as strategic thinking and leadership. This study would inform targeted educational strategies that enhance research capacity and leadership skills, ultimately advancing the pharmacy profession in both academic and practice settings.

## 2 Methods

### 2.1 Study design

A cross-sectional descriptive and analytical study was conducted from December 2023 to May 2024 to evaluate research self-efficacy related to leadership, general self-efficacy, and strategic thinking among early-career pharmacists in Lebanon, including pharmacy students and recent graduates.

### 2.2 Procedure and sampling

Data were collected through an online questionnaire created on Google Forms. The link was distributed to all pharmacy students and other early-career pharmacists who graduated within the past decade, as defined by the Pharmaceutical Society of Australia.^20^ Distribution channels included emails and relevant university groups through the Lebanese Pharmacy Students Association (LPSA); the dissemination was repeated through the same channels until the minimum sample size was reached. This census-based approach yielded voluntary participation, and the resulting dataset represents a self-selected convenience sample.

### 2.3 Data collection tool

The questionnaire was pilot-tested for clarity among 10 individuals; the final version was adopted after correcting few typographical mistakes. It was administered in English and consisted of two sections (https://forms.gle/5Y5h9DwYGSUsMwGo7). The first section gathered sociodemographic and education-related variables, while the second included standardized scales measuring research-related key constructs.

#### 2.3.1 The Research Self-Efficacy Scale

The Research Self-Efficacy Scale (RSES)-Short Version^21^^,^^22^ needs validation before use in Lebanon. The RSES is generally used to assess participants' confidence in their ability to perform research tasks. The used version consists of five items, with total scores ranging from 5 to 25.^21^^,^^22^ This ultra-short form captures core elements of research competence while minimizing respondent burden. The items assess self-efficacy across foundational research domains, including: identifying and articulating a research problem, selecting or applying appropriate methodological approaches, analyzing and interpreting data, integrating research findings into coherent conclusions, and communicating results effectively. Higher total scores indicate greater perceived capability to carry out key components of the research process. Despite its brevity, the 5-item version has shown strong psychometric performance in prior applications, with Cronbach's alpha values commonly reported above 0.80, supporting its reliability for assessing overall research-related self-efficacy.^21^^,^^22^ This scale was used as is (without linguistic adaptation) and was validated before its results were adopted.

#### 2.3.2 Other previously validated tools

These scales were previously validated in Lebanon and used to assess leadership, general self-efficacy, and strategic thinking, as follows^23^:

• The Authentic Leadership Self-Assessment Questionnaire (ALSAQ) evaluated leadership skills among participants. This 16-item instrument is grounded in Authentic Leadership Theory and organized into four subscales: self-awareness, internalized moral perspective, balanced processing, and relational transparency. Each item is rated on a Likert-type scale, enabling quantification of authentic leadership behaviors; it has an appropriate internal consistency (Cronbach alpha = 0.84). It helps identify both strengths and weaknesses in leadership performance.^24^
• The general self-efficacy was measured using the widely used General Self-Efficacy Scale (GSES), a 10-item instrument with total scores ranging from 10 to 40. The scale is designed to measure individuals' global confidence in their ability to cope with a wide range of demanding or novel situations. Higher scores reflect participants' increased ability to handle challenging situations effectively. The GSES has demonstrated strong internal consistency (commonly α = 0.80–0.90 across cultures) and robust construct validity, making it suitable for use in health professions research, education, and behavioral studies.^25^
• The Strategic Thinking Questionnaire (STQ) assessed strategic thinking capabilities through 15 items, with higher scores indicating more robust strategic thinking skills. This 15-item instrument captures multiple cognitive processes involved in strategic behavior, including visioning, systemic thinking, futuristic/long-term orientation, and intent-focused decision-making. Respondents rate each item on a Likert-type scale, and total scores reflect the overall strength of their strategic thinking abilities. The STQ demonstrated acceptable psychometric properties in prior studies (internal consistency estimates typically above 0.80), making it suitable for evaluating strategic cognition in professional and educational settings.^14^

### 2.4 Minimum sample size calculation

The minimum sample size was calculated using the G-Power software (version 3.0.10). The calculation was based on an expected effect size of 0.0526 and a squared multiple correlation R^2^ of 0.05 for the omnibus test of multiple regression. The minimum required sample size was 454 participants, considering a statistical power of 80 %, an alpha error of 5 %, and 25 predictors to be included in the model. A minimal sample of 500 participants was targeted to account for potential missing data and ensure robust validation (a minimum of ten participants per item).

### 2.5 Ethical aspects

The INSPECT-LB Research Ethics Committee approved the study protocol (approval number: 2023REC-013-INSPECT-10-09). Participants were informed about the study's objectives and its voluntary nature, with the option to withdraw at any time. Anonymity and data confidentiality were maintained throughout data collection and analysis. Participants provided informed consent before proceeding to the survey.

### 2.6 Statistical analysis

Data analysis was performed using Statistical Package for Social Sciences (SPSS) software, version 28.0. The unvalidated scale (RSES) was first validated using exploratory factor analysis,^26^ with sampling adequacy assessed using the Kaiser-Meyer-Olkin measure and the Bartlett test for sphericity.^27^ Structural validity was evaluated using Spearman correlation coefficients to correlate between items, factors, and the overall score. A strong correlation was considered whenever *r* > 0.5.^28^ The score reliability (internal consistency) was assessed using Cronbach's alpha.^29^

For descriptive analysis, frequency and percentage were used for categorical variables, while means and standard deviations, as well as medians and interquartile regions, were calculated for quantitative variables. The distribution of quantitative variables was assessed for normality by visual inspection of the histogram, with skewness and kurtosis below 1. These conditions, combined with a sample size exceeding 300, were considered compatible with normal distribution.^30^

Bivariate analysis of continuous variables was performed using the Student's *t*-test to compare means between two groups and ANOVA for comparisons across three groups or more after verification of variance homogeneity with Levene's test. When variances were not homogeneous, the corrected t-test and the Kruskal-Wallis test were applied, respectively. Post hoc analyses were conducted with Bonferroni adjustment for significant results in ANOVA or Kruskal-Wallis tests. Spearman correlation coefficients were used to examine associations between continuous variables. For categorical variables, the Chi-squared test was applied, with the Fisher exact test used when expected values were below 5. In all cases, a *p*-value lower than 0.05 was considered statistically significant.

For the multivariable analysis, multiple linear regressions were conducted to assess the correlates of the dependent variables in the entire sample. Assumptions, including normality of residuals, linearity, the absence of multicollinearity, and homoscedasticity, were verified before analysis. The ENTER method with all independent variables was used to present the most comprehensive model, reporting the beta coefficient, its 95 % confidence interval, and the *p*-value.^31^

## 3 Results

### 3.1 Sample characteristics

The study included 504 early-career pharmacists: 68 % students and 32 % recent graduates. The majority were female (71 %) and single (86.1 %), with a mean age of 23.81 years.

Regarding personal monthly income, 38.1 % reported no income, while 12.5 % earned less than 250 USD, 22.2 % earned 250–500 USD, 19.6 % earned 500–1000 USD, and 7.5 % earned more than 1000 USD. Geographically, participants were predominantly from Beirut (39.9 %), followed by Mount Lebanon (18.1 %) and the South (17.1 %). Regarding university distribution, 48.6 % were enrolled at LIU, 20.4 % at BAU, and 19 % at LU. Around one-third (32 %) were graduates, 30 % were senior students, and the remainder were fourth-year students or below. Educational attainment showed that 55 % were undergraduate students, 23.2 % held a bachelor's degree, 11.7 % had a PharmD, 9.3 % held a master's degree, and 0.6 % had a doctorate. Regarding employment, 25.4 % were full-time employees, 22.4 % were part-time employees, 8.1 % were self-employed, and 44 % did not work.

### 3.2 Factor analysis of the Research Self-Efficacy Scale Short Form (RSES-11)

The factor analysis of the RSES-11 demonstrated excellent construct validity, with all items loading onto one single factor that accounted for 65.4 % of the variance. Factor loadings ranged from 0.666 to 0.876, indicating strong inter-item and item-construct correlations. The scale exhibited excellent internal consistency, with a Cronbach's alpha of 0.946. Sampling adequacy was confirmed by a Kaiser-Meyer-Olkin (KMO) value of 0.943, and Bartlett's test of sphericity was significant (*p* < 0.001), further supporting the scale's validity (Table 1). RSES scores ranged from a 25th percentile of 61 to a median of 73.5 (equivalent to 65/100) and a 75th percentile of 84.

**Table 1:** Factor analysis of the Research Self-Efficacy Scale short form (RSES-11).

	Factor loading
7. Design and implement the best strategy for collecting my data	0.876
5. Choose a research design that will answer my research question or hypothesis	0.867
8. Design and implement the best data analysis strategy for my research study	0.864
10. Teach someone else how to design and implement a simple research project	0.828
9. Effectively present my study and its implications to large groups of clinical/public health researchers	0.823
4. Write a balanced and comprehensive literature review	0.815
6. Put together a team to help me to conduct my research	0.805
11. Write a scientific report out of my results research	0.802
2. Formulate a clear research question or a testable hypothesis to address a clinical/public health problem	0.780
3. Do an effective electronic database search of the literature	0.747
1. Identify a clinical problem that is amenable to research	0.666
Percentage variance explained	65.40 %
Cronbach alpha = 0.946	
Kaiser-Meyer-Olkin (KMO) = 0.943	
Bartlett's test of sphericity *p* < 0.001	

### 3.3 Bivariate analysis of the RSES scores

Participants had slightly above-average mean scores across all concepts, including research self-efficacy, except for generalized self-efficacy, where scores fell below average. Overall, RSES distribution was normal across all subgroups. Participants with the lowest income (<250 $US) had a significantly lower RSES score, while those from the LAU and foreign universities (outside Lebanon) had significantly higher scores. Moreover, RSES was moderately correlated with leadership, global self-efficacy, and strategic thinking (*p* < 0.001) (Table 2).

**Table 2:** Bivariate analysis of the sample (*N* = 504).

Multinomial and dichotomous variables	Frequency (%)	*P*-value Mean RSES (SD)
Gender		0.063
Male	146 (29.0 %)	74.80(17.65)
Female	358 (71.0 %)	71.62 (17.29)
Marital status		0.366
Single	434 (86.1 %)	72.17 (17.40)
Married	66 (13.1 %)	75.15 (17.61)
Divorced	3 (0.6 %)	76.00 (19.08)
Widowed	1 (0.2 %)	
Personal monthly income		0.002
No income	192 (38.1 %)	78.83 (17.82)
<250 USD ($)	63 (12.5 %)	66.90 (14.92)⁎
250–500$	112 (22.2 %)	75.38 (17.34)
500–1000$	99 (19.6 %)	73.83 (17.38)
>1000$	38 (7.5 %)	78.76 (16.84)
Region of living		0.188
Beirut	201 (39.9 %)	71.48 (17.45)
Mount Lebanon	91 (18.1 %)	72.58 (15.54)
North	58 (11.5 %)	74.86 (19.37)
South	86 (17.1 %)	75.61 (17.77)
Beqaa	68 (13.5 %)	69.77 (17.39)
Current/graduation university		0.002
LU	96 (19.0 %)	72.36 (15.43)
USJ	25 (5.0 %)	72.08 (22.35)
BAU	103 (20.4 %)	76.11(17.64)
LAU	14 (2.8 %)	81.00 (11.65)⁎
LIU	245 (48.6 %)	69.89 (16.58)
Foreign university (outside Lebanon)	21 (4.2 %)	81.71 (24.47)⁎
Current academic year		0.499
First	11 (2.2 %)	67.09 (26.71)
Second	18 (3.6 %)	74.83 (12.75)
Third	36 (7.1 %)	69.33 (17.70)
Fourth	124 (24.6 %)	71.45 (17.83)
Fifth	135 (26.8 %)	72.31 (16.32)
Sixth	16 (3.2 %)	77.25 (14.81)
Graduate student	89 (17.7 %)	72.63 (19.71)
Not studying anymore	75 (14.9 %)	75.45 (15.56)
Highest level of education achieved		0.260
I am an undergraduate student	277 (55.0 %)	72.91 (17.09)
Bachelor's degree	117 (23.2 %)	71.37 (17.20)
PharmD	59 (11.7 %)	75.44 (17.07)
Master's degree (MBA, MPH, etc.)	47 (9.3 %)	73.44 (19.51)
Doctorate (PhD, DBA, etc.)	3 (0.6 %)	87.33 (20.50)
Employment status		0.098
Full-time employee	128 (25.4 %)	74.47 (16.90)
Part-time employee	113 (22.4 %)	72.37 (17.34)
Self-employed	41 (8.1 %)	76.73 (14.52)
I do not work	222 (44.0 %)	70.75 (18.14)
Scale variables	Mean ± SD	Correlation (*p*-value)
Age (in years)	23.81 ± 4.30	0.048 (0.279)
Authentic Leadership Self-Assessment Questionnaire (ALSAQ-16) (Max /90)	57.26 ± 11.03	0.273 (<0.001)
Global Self-Efficacy Questionnaire (GSE-10) (Max /50)	20.32 ± 5.73	0.360 (<0.001)
Strategic Thinking Questionnaire (STQ-15) (Max/75)	51.63 ± 11.66	0.322 (<0.001)
Research Self-Efficacy Scale (RSES-11) (Max/110)	72.54 ± 17.44	–

Abbreviations: ALSAQ: Authentic Leadership Self-Assessment Questionnaire; BAU: Beirut Arab University; DBA: Doctor of Business Administration; DPT: Doctor of Physical Therapy; GSES: General Self-Efficacy Scale; LAU: Lebanese American University; LIU: Lebanese International University; LU: Lebanese University; MBA: Master of Business Administration; MPH: Master of Public Health; PharmD: Doctor of Pharmacy; PhD: Doctor of Philosophy; RSES: Research Self-Efficacy Scale; SD: Standard Deviation; STQ: Strategic Thinking Questionnaire; USD: United States Dollar; USJ: Saint Joseph University.

⁎ Asterisks indicate categories that differ significantly from the other categories within a given variable (*p* < 0.05).

### 3.4 Multivariable analysis

The multivariable analysis identified several factors associated with research self-efficacy among early-career pharmacists. Participants with a lower income (<250 US$) had significantly lower research self-efficacy compared to those with higher incomes (> 500 US$) (B = −5.016; *p* = 0.030). Conversely, those living in the South versus Beirut (B = 4.913; *p* = 0.022) and attending a foreign university (outside Lebanon) compared to the LU (public university) (B = 9.275; *p* = 0.039) had higher research self-efficacy. Higher scores on the GSE scale (B = 0.582; *p* < 0.001) and the STQ (B = 0.215; *p* = 0.005) were also associated with higher research self-efficacy (Table 3).

**Table 3:** Research Self-Efficacy (RSES) correlates among early-career pharmacists.

Parameter	B	Sig.	95 % Confidence Interval	
			Lower Bound	Upper Bound
Marital status (not single versus single)	1.408	0.562	−3.361	6.177
Income				
Low < 250$ vs High > 500$	−5.016	0.030	−9.540	−0.491
Middle 250–500$ vs High >500$	0.340	0.877	−3.980	4.659
Gender				
Female versus Male	−1.893	0.256	−5.168	1.381
Region of Living				
Beqaa region vs Beirut	1.578	0.510	−3.125	6.281
Mount Lebanon vs Beirut	1.008	0.627	−3.062	5.079
North vs Beirut	4.887	0.051	−0.028	9.803
South vs Beirut	4.913	0.022	0.703	9.124
University (Private versus Public)				
BAU vs LU (Public University)	2.987	0.233	−1.930	7.904
Foreign vs LU (Public University)	9.275	0.039	0.469	18.082
LAU vs LU (Public University)	7.503	0.119	−1.934	16.939
LIU vs LU (Public University)	−2.070	0.348	−6.399	2.259
USJ vs LU (Public University)	−0.864	0.837	−9.120	7.392
Current academic year				
First vs Not studying anymore	3.848	0.560	−9.112	11.201
Second vs Not studying anymore	8.742	0.117	−2.189	19.674
Third vs Not studying anymore	5.008	0.245	−3.444	13.459
Fourth vs Not studying anymore	3.815	0.311	−3.571	11.201
Fifth vs Not studying anymore	5.294	0.129	−1.541	12.129
Sixth vs Not studying any more	0.736	0.874	−8.362	9.834
A graduate student vs Not studying anymore	−3.039	0.255	−8.284	2.205
Scales				
ALSAQ	0.151	0.082	−0.019	0.321
GSE	0.582	<0.001	0.275	0.889
STQ	0.215	0.005	0.064	0.366
Age in years	0.056	0.828	−0.448	0.560
The highest level of education achieved				
Bachelor's degree versus Undergraduate	0.551	0.834	−4.629	5.731
Doctorate (PhD, DBA) versus Undergraduate	16.099	0.117	−4.066	36.264
Master's degree (MBA, MPH, MS) versus Undergraduate	4.890	0.219	−2.920	12.700
PharmD versus Undergraduate	4.891	0.203	−2.643	12.425

Abbreviations: Foreign: Outside Lebanon; ALSAQ: Authentic Leadership Self-Assessment Questionnaire; BAU: Beirut Arab University; DBA: Doctor of Business Administration; GSES: General Self-Efficacy Scale; LAU: Lebanese American University; LIU: Lebanese International University; LU: Lebanese University; MBA: Master of Business Administration; MPH: Master of Public Health; MS: Master of Sciences; PharmD: Doctor of Pharmacy; PhD: Doctor of Philosophy; RSES: Research Self-Efficacy Scale; STQ: Strategic Thinking Questionnaire; USD: United States Dollar; USJ: Saint Joseph University.

## 4 Discussion

The present study confirmed the validity of the Research Self-Efficacy Scale (RSES) and assessed research self-efficacy among early-career pharmacists in Lebanon, in association with pharmacists' personal attributes. The RSES exhibited excellent psychometric properties, loading on a single factor with strong validity and reliability. These results align with previous findings from studies validating the scale across diverse populations, confirming its robustness in different contexts.^32^

Findings revealed that pharmacists who attended universities outside Lebanon had higher RSES scores compared to those from Lebanese institutions. However, no significant differences were observed among the various Lebanese faculties. This disparity may reflect the enhanced educational environments and resources available at foreign institutions, often providing greater exposure to research opportunities and fostering the development of research skills, particularly in high-income countries.^33^^,^^34^ Previous research has demonstrated that high-quality education and access to diverse research experiences significantly influence self-efficacy, indicating that a well-structured educational context is essential for developing research competencies.^35^ Moreover, the institutional training environment, including research-focused curricula and opportunities for both faculty and student development, plays a critical role in building research self-efficacy.^36^ Lebanese universities should strengthen their educational frameworks by integrating more comprehensive research opportunities and support systems, revising curricula to include more research-oriented courses, fostering international collaborations and exchange programs, enhancing faculty research abilities, and providing faculty with the necessary resources to mentor students effectively. Initiatives such as the National Pharmaceutical Research Strategy (1) advocate for such environments, aiming to improve professional readiness in research among pharmacy graduates.^15^^,^^21^^,^^37^, ^38^, ^39^

This study revealed a positive correlation between higher research self-efficacy scores and higher GSE, STQ, and ALSAQ scores, consistent with existing literature that presents the complex interplay between research skills and personal characteristics. Generalized self-efficacy reflects an individual's confidence in their ability to perform tasks and achieve goals, which is crucial for engaging in research activities.^25^ Strategic thinking, defined as the ability to anticipate environmental conditions and shape a promising future distinct from the present,^13^^,^^14^ enables pharmacists to define and visualize outcomes, align their work with the evolving needs of pharmacy practice, and ensure the relevance and impact of their research.^13^^,^^14^ Pharmacy curricula should include exposure to the evolving role of pharmacists to support the development of strategic thinking and self-efficacy. Frameworks, such as the 9-star pharmacist model,^40^ the development goals outlined by the International Pharmaceutical Federation (FIP),^41^ and the global 2030 roadmap,^42^ provide valuable guidance. Integrating these concepts into pharmacy education could help students understand the diverse and dynamic roles they are expected to fulfill in many fields, including research. Additionally, emphasizing modern aspects of pharmacy practice, such as leadership, patient-centered care, and interprofessional collaboration, can encourage students to think critically about the future of pharmacy. This approach would foster a sense of responsibility and adaptability among students while enhancing their self-efficacy by equipping them with the strategic mindset needed to excel in research and professional practice.

Interestingly, while differences in RSES scores were observed across academic levels and years, consistent with previous findings,^43^ these differences did not reach statistical significance. This outcome is likely due to the small sample sizes within subgroups, such as doctorate candidates, or slight differences between academic levels, which may have reduced the statistical power to detect significant differences. This result suggests that individual motivation and institutional support are more critical in influencing research self-efficacy than academic level or experience alone.

Finally, this study showed that early-career pharmacists with a lower income had significantly lower research self-efficacy. Although no prior studies have examined this correlation in research self-efficacy, evidence from other domains indicates that socioeconomic status may affect both academic and career self-efficacy, with female students from lower SES groups having lower levels of self-efficacy than their higher-SES counterparts when adjusting to college environments.^44^ These findings indicate that targeted interventions are essential to supporting early-career professionals and students from diverse backgrounds and enhancing their research self-efficacy.

### 4.1 Limitations and strengths

Several limitations must be acknowledged in this study. The cross-sectional design limits the ability to establish causality between participants' characteristics and research self-efficacy. Additionally, the use of electronic platforms for questionnaire dissemination may have introduced selection bias, particularly given the variability in internet access in Lebanon and differences in digital literacy between participants and those who opted not to participate. The reliance on self-reported data raises concerns about social desirability bias, where respondents might have overestimated their competencies. Moreover, the questionnaire's length could have led to respondent fatigue, potentially resulting in non-differential information bias and skewing results toward the null hypothesis.

Despite these limitations, the study has several notable strengths. Its diverse sample from all universities minimizes the risk of selection bias, since the presented percentages can be considered similar to the ones available from the OPL.^45^ The use of validated scales enhances the validity of the findings and reduces the risk of information bias. The application of multivariable analysis also minimizes confounding bias, adding robustness to the results. Most importantly, this study is the first to correlate research self-efficacy in pharmacists with various personal characteristics using validated scales. Future research using longitudinal designs could provide valuable insights into causal relationships. Such prospective studies could offer a better understanding of the factors influencing research self-efficacy among early-career pharmacists, paving the way for targeted interventions to enhance research capabilities within this population.

## 5 Conclusion

In conclusion, this study demonstrated that the RSES is a valid and reliable tool for assessing research self-efficacy among early-career pharmacists in Lebanon. The findings revealed that pharmacists from foreign universities scored higher on the RSES compared to their counterparts from Lebanese institutions. Additionally, a positive correlation was found between research self-efficacy, generalized self-efficacy, strategic thinking, and leadership scores. Several strategies could be implemented at the institutional and national levels to strengthen self-efficacy and cultivate a sustainable research environment. These include enhancing educational frameworks, integrating research opportunities, and fostering international collaborations. Future research using this validated scale will be instrumental in evaluating the effectiveness of such interventions.

## CRediT authorship contribution statement

**Aline Hajj:** Writing – original draft, Methodology. **Hala Sacre:** Writing – review & editing, Validation. **Chadia Haddad:** Writing – review & editing, Formal analysis, Data curation. **Jenny Elia:** Writing – review & editing, Project administration. **Joya El Ghawi:** Writing – review & editing, Project administration. **Lina Haidar:** Writing – review & editing, Project administration. **Lama Dimachkieh:** Writing – review & editing, Project administration. **Mahmoud Nasrallah:** Writing – review & editing, Project administration. **Jihan Safwan:** Writing – review & editing. **Deema Rahme:** Writing – review & editing. **Sukaina Basma:** Writing – review & editing. **Salameh Pascale:** Writing – review & editing, Validation, Supervision, Conceptualization.

## Funding

No funding was received for conducting this study.

## Declaration of competing interest

I have nothing to declare.
